# Wood cookstove use is associated with gastric cancer in Central America and mediated by host genetics

**DOI:** 10.1038/s41598-023-42973-7

**Published:** 2023-10-02

**Authors:** Samara B. Rifkin, Anna K. Miller, Eleazar E. Montalvan-Sanchez, Dalton A. Norwood, Enrique Martinez, Tim Waterboer, T. Mark Beasley, Ricardo L. Dominguez, Scott M. Williams, Douglas R. Morgan

**Affiliations:** 1 Department of Gastroenterology and Hepatology, University of Michigan, Ann Arbor, MI USA; 2 Department of Genetics and Genome Sciences, Case Western Reserve University, Cleveland, OH USA; 3 Hospital de Occidente, Ministry of Health, Santa Rosa de Copan, Copan, Honduras; 4 Department of Medicine, Indiana University School of Medicine, Indianapolis, IN USA; 5 Division of Preventive Medicine, Department of Medicine, The University of Alabama at Birmingham, Birmingham, AL USA; 6 Hospital Evangelico, Siguatepeque, Honduras; 7 Infections and Cancer Epidemiology, German Cancer Research Center (DKFZ), Heidelberg, Germany; 8 Department of Biostatistics, School of Public Health, The University of Alabama at Birmingham, Birmingham, AL USA; 9 Birmingham/Atlanta Geriatric Research, Education, and Clinical Center, Birmingham Veterans Affairs Medical Center, Birmingham, AL USA; 10 Department of Population and Quantitative Health Sciences and Cleveland Institute for Computational Biology, Case Western Reserve University, Cleveland, OH USA; 11 Medicine and Epidemiology, UAB Division of Gastroenterology and Hepatology, The University of Alabama at Birmingham (UAB), Birmingham, AL 35294 USA

**Keywords:** Gastric cancer, Environmental impact, Pathogens, Cancer genetics, Genetic association study

## Abstract

Biomass cookstove food preparation is linked to aero-digestive cancers, mediated by ingested and inhaled carcinogens (e.g., heterocyclic amines, and polycyclic aromatic hydrocarbons). We investigated the association between gastric adenocarcinoma, wood cookstove use, *H. pylori* CagA infection and risk modification by variants in genes that metabolize and affect the internal dose of carcinogens. We conducted a population-based, case–control study (814 incident cases, 1049 controls) in rural Honduras, a high-incidence region with a homogeneous diet and endemic *H. pylori* infection, primarily with the high-risk CagA genotype. We investigated factors including wood cookstove use, *H. pylori* CagA serostatus, and 15 variants from 7 metabolizing genes, and the interactions between wood stove use and the genetic variants. Male sex (OR 2.0, 1.6–2.6), age (OR 1.04, 1.03–1.05), wood cookstove use (OR 2.3, 1.6–3.3), and CagA serostatus (OR 3.5, 2.4–5.1) and two SNPs in *CYP1B1* (rs1800440 and rs1056836) were independently associated with gastric cancer in multivariate analysis. In the final multivariate model, a highly significant interaction (OR 3.1, 1.2–7.8) was noted between wood cookstove use and the rs1800440 metabolizing genotype, highlighting an important gene-environment interaction. Lifetime wood cookstove use associates with gastric cancer risk in the high-incidence regions of Central America, and the association is dependent on the rs1800440 genotype in *CYP1B1*. *H. pylori* CagA infection, wood cookstove use and the rs1800440 genotype, all of which are highly prevalent, informs who is at greatest risk from biomass cookstove use.

## Introduction

Gastric adenocarcinoma (GC) is the leading cause of infection-related cancer mortality globally, and overall is the third leading cause of cancer death^1–5^. One million incident cases occur annually, and high-incidence regions include Latin America, eastern Asia, and eastern Europe. In Latin America, a large burden of disease is concentrated in the mountainous regions along the Pacific littoral^6^. This pattern has been described as the gastric cancer “altitude enigma”, and suggests a clustering of risk exposures in the rural mountain villages^6,7^. *Helicobacter pylori* (*H. pylori*) infection is the primary risk factor for gastric cancer, acting in concert with host genetics and responses, dietary factors, and environmental exposures^8^. *H. pylori* genetic variation affects risk, and CagA is the principal *H. pylori* virulence genotype for gastric adenocarcinoma^8^.

Cooking and heating with biomass fuel is an environmental risk for a variety of cancers. One-third of the world’s population burns organic matter (wood, charcoal, dung) for domestic energy, leading to multiple health and environmental consequences^9^. The mechanisms by which biomass cooking and heating increases cancer risk is hypothesized to be exposures to organic carcinogens, such as heterocyclic amines (HCAs), and inhaled airborne pollutants, including polycyclic aromatic hydrocarbons (PAHs)^10^. The effects of this exposure is exacerbated by poor ventilation in the mountainous regions^11^. Metal carcinogens from the cooking surface may also contribute to risk^12,13^. Red meat cooked on biomass stoves contribute to cancer risk by the ingestion of carcinogens, including HCAs and PAHs, created during cooking at high temperatures^14^. HCAs are formed when amino acids, sugars, and creatine in meat react at high cooking temperatures, while PAHs are formed when fat vaporizes and then adheres to the surface of the meat^15^. High temperature cooking of other foods may also introduce risk^16^. The International Agency for Research on Cancer (IARC) has therefore classified red and processed meat as a Class I carcinogen, linked to the increased relative risk of colon cancer, gastric cancer, and other cancers^14^.

The relationship between exposure to HCAs and PAHs and gastric cancer may be modified by genetic variation in genes encoding enzymes that activate or deactivate HCAs and PAHs^17–22^. These enzymes include phase I enzymes, such as CYP1A2 and CYPB1, and phase II enzymes, including N-acetyltransferases (NAT) and glutathione S transferases (GSTs). Phase I enzymes tend to activate HCAs and PAHs into carcinogenic metabolites that create DNA adducts and lead to mutations, whereas some phase II enzymes can detoxify carcinogenic compounds and prevent further damage^17^. Finally, the Aryl hydrocarbon receptor (AHR) can mediate expression of both classes of enzymes. These activating and detoxifying enzymes in combination may determine the internal exposure (“internal dose”) to environmental carcinogens, and hence modify cancer risk due to environmental exposures^17^. Polymorphisms of enzymes encoding genes that increase the activity or level of phase I enzymes and decrease the activity or level of phase II enzymes can lead to a higher risk of cancer^17^.

Wood cookstove (“fogon”) use is common in the mountainous regions of Pacific Latin America, where gastric cancer incidence is amongst the highest in the world, and cookstove use may contribute to the excess burden of gastric cancer. In the Central America Four (CA-4, Honduras, Guatemala, El Salvador, Nicaragua) region, wood stove use is the predominant method of cooking and *H. pylori* infection is endemic (70–90% of the adult population). The CA-4 countries comprise the core low/middle income country (LMIC) region in the western hemisphere, with a population of over 40 million, and is linked to an immigrant population in the U.S. of 6 million^6,23^.

We hypothesized that wood cookstove use increases the risk of gastric adenocarcinoma and that the risk is modified by HCA- and PAH- metabolizing enzyme genotypes. The effects of wood stove use exposures may be most discernable in populations with homogeneous diets and external HCA-exposures, and endemic high-risk *H. pylori* infection (e.g., CagA genotype), as in the rural populations of Central America^24,25^.

## Materials and methods

### Study design

We conducted a population-based, case–control study based in the mountainous regions of Honduras. This region has among the highest incidence rates in the western hemisphere, with a high prevalence of chronic *H. pylori* infection (80–90%)^25–29^. Incident GC cases were enrolled prospectively from the two district hospitals (Santa Rosa de Copán and Siguatepeque) that serve the mountainous rural areas of west-central Honduras. The diagnosis of GC was based on endoscopic appearance and confirmatory histopathology. Household interviews were conducted for randomly selected healthy controls from a wide distribution of villages in the region and the catchment area, as described previously^25,28^. Once consented, patients and control subjects would undergo the study interview with review of exclusionary health criteria, as well as the demographic, health assessment, and epidemiology questionnaires. Lifetime woodstove use was dichotomized (yes, no) since we observed minimal lifetime variation in usage nor transition to other types of cook stoves in the Honduras rural populations.

### *H. pylori* infection and CagA assessment

A validated multiplex serology was used to determine *H. pylori* and CagA serostatus, the dominant bacterial risk genotype for GC^30–32^. We focused on CagA, the principal oncoprotein, as the region has an extremely high, *H. pylori* prevalence. The multiplex serology panel was developed by the Germany Cancer Research Center (“DKFZ”). In brief, the *H. pylori* proteins were recombinantly expressed as Glutathione-S-transferase (GST)-tag fusion proteins in *Escherichia coli* BL21 and affinity-purified on glutathione-coated fluorescently labeled polystyrene beads (Luminex Corp.). A mixture of the differently labeled and antigen-loaded beads was incubated with serum to allow binding of serum antibodies to the *H. pylori* proteins. Bound serum antibodies were detected by a biotin-labeled anti-human IgM/IgA/IgG secondary antibody and Streptavidin-R-phycoerythrin. The Luminex 200 analyzer (Luminex Corp.) distinguished between the bead type and the bound antigen and quantified the amount of bound serum antibody as median fluorescence intensity (MFI) of 100 beads per type measured. The CagA antigen-specific cutoff was used^30^.

### Candidate genes

We selected 15 variants from 7 HCA metabolizing genes previously associated with colorectal polyp risk^17^ (Table S1). The genes and single nucleotide polymorphisms (SNPs) of interest include: Epoxide Hydrolase 1 (*EPHX1*) (rs1051740), Cytochrome P450 Family 1 Subfamily B Member 1 (*CYP1B1*) (rs1800440 and rs1056836), Aryl hydrocarbon receptors (*AHR*) (rs2066853), N-Acetyltransferase 1 (*NAT1*) (rs1799931, rs15561, rs1208, 1799930, rs1041983, rs1799929, rs1801279, and rs1801280), Cytochrome P450 Family 2 Subfamily E Member 1 (*CYP2E1*) (rs2031920), UDP Glucuronosyltransferase Family 1 Member A7 (*UGT1A7*) (rs61261057) and Cytochrome P450 Family 1 Subfamily A Member 2 (*CYPIA2*) (rs762551). Primers for each of these SNPs are presented in Table S2.

### Genotype analysis

Human DNA was isolated from whole blood samples with the Qiagen Puregene® kit and genotyped on the MassARRAY® Sequenom platform at Vanderbilt University Medical Center (VANTAGE Core facility). We tested for Hardy–Weinberg equilibrium and removed SNPs that deviated in cases and controls at p < 1.0 × 10^–6^. We also removed SNPs that had a minor allele frequency (MAF) < 0.05 using PLINK (version 1.9)^33–35^. We tested for Linkage Disequilibrium in PLINK and Haploview (version 4.2) regardless of case–control designation to determine the number of independent tests for the False Discovery Rate (FDR) threshold and limit multiple testing^36^.

### Statistical analysis

We compared differences between case and control subjects using t-tests for the continuous variable age or the chi-square test for the categorical variables sex, wood stove use, and bacterial CagA serostatus. Age was tested for normality using the Shapiro–Wilk’s method with the R “stats” package (R version 4.0.4). Age was negatively skewed as GC is the final step of progressive gastric disease, and the population was predominately older^37^. Therefore, age was log transformed, and the analyses were run with the transformed variable, but the deviation from a normal distribution did not impact the results substantively. The untransformed age variable is presented in all the results.

Univariate logistic regression models were used to estimate risk of GC associated with individual variables, including all SNPs. Logistic regression was used to estimate odds ratios (OR) for adjusted models that included combinations of the variables age, sex, CagA serostatus, wood stove use, and each SNP individually. Daily tobacco use was very low (20 pack-year history, 4.8%), and was not included in the model. For each SNP, we also assessed multivariable models for individuals without missing data that included an interaction term with wood stove use. A likelihood ratio test using R package “lmtest” was used to assess the goodness of fit of competing statistical models, i.e., those without the interaction and those with the interaction (R version 4.0.4). The R command “p.adjust” was used to calculate the q-values using the Benjamini–Hochberg test with a False Discovery Rate (FDR) level of 0.1 for all tests (R version 4.0.4). We confirm that all research was performed in accordance with relevant guidelines/regulations and informed consent was obtained from all participants and/or their legal guardians. Research involving was performed in accordance with the Declaration of Helsinki.

### Ethics committee approvals

The study was approved by the institutional review boards of The University of Alabama at Birmingham, Vanderbilt University and the Ministry of Health in western Honduras.

### Consent to participate

Informed consent was obtained from all individual participants included in the study.

## Results

A total of 814 gastric cancer patients and 1049 population controls were enrolled, of whom genetic and *H. pylori* data were available for 1,425 participants (Table 1). The median ages of the cases and controls were 65 and 54, respectively. Approximately one-fifth of cases were under the age of 55 (n = 170 of 814). Overall, 87% and 85% of subjects were positive for *H. pylori* infection and CagA, respectively. Wood stove use was observed in 92% of the gastric cancer cases and in 78% of the population-based controls. Gastric cancer cases were more likely to be older, male, have lifetime wood stove use, and be positive for CagA serostatus. In univariate analyses, age (β = 1.04, p < 2.00 × 10^–16^), sex (OR = 2.51, p = 2.53 × 10^–16^), wood stove use (OR = 2.57, p = 5.52 × 10^–8^), and bacterial CagA serostatus (OR = 3.51, p = 4.20 × 10^–13^) all associated with an increased risk of gastric cancer (Table 2). In a multivariate model including age, sex, wood stove use and bacterial CagA serostatus, these variables all remained significantly associated with gastric cancer.

**Table 1:** The characteristics of the gastric cancer cases and population controls.

Characteristics	Control population^a^ n (%)	Gastric cancer cases^a^ n (%)	P-value
Age			
Mean (SD)	54.3	64.1	< 0.0001
Sex			
Female	550 (49.7)	260 (31.9)	< 0.0001
Male	557 (50.3)	554 (68.1)	
Wood stove use			
Yes	784 (77.9)	713 (91.9)	< 0.0001
No	222 (22.1)	61 (7.9)	
*H. pylori* serostatus			
Yes	677 (87.4)	568 (87.5)	0.93
No	98 (12.6)	81(12.5)	
CagA serostatus			
Yes	605 (78.1)	600 (92.5)	< 0.0001
No	170 (21.9)	48 (7.5)	

^a^There were 814 gastric cancer patients and 1,049 population controls enrolled; Complete genotyping and *H. pylori* serology data were available for 1,425 participants.

**Table 2:** Univariate and multivariate odds ratios for gastric cancer outcomes.

Characteristics	Univariate OR (95% CI)	Multivariate OR (95% CI)^a^
Age	1.04 (1.03, 1.05)	1.04 (1.03, 1.05)
Sex		
Male	2.51 (2.02, 3.13)	1.99 (1.56, 2.55)
Female	Referent	Referent
Wood stove use		
Yes	2.57 (1.84, 3.64)	2.33 (1.62, 3.39)
No	Referent	Referent
Bacterial CagA serostatus		
Yes	3.51 (2.52, 4.98)	3.49 (2.41, 5.13)
No	Referent	Referent

*95% CI* 95% confidence interval.

^a^Multivariate model includes age, sex, wood stove use, and CagA positive serostatus.

Of the 15 SNPs genotyped, none were out of Hardy–Weinberg equilibrium (p < 1.0 × 10^–6^ for both cases and controls), but 2 SNPs had a minor allele frequency of less than 0.05 and were excluded from further analyses (Table S1). Allele frequencies of the SNPs were within the expected ranges, based on the Latin American populations in the 1000 Genomes databases (Version 3). Linkage Disequilibrium (L.D.) was tested for the 6 SNPs in the NAT1 gene that met inclusion criteria. Three pairs showed evidence of significant correlation (r^2^ > 0.4), and we randomly removed one SNP from each pair to reduce the multiple testing burden (Fig. S1). The data cleaning resulted in 10 SNPs being tested in the final analysis. Genotype counts are presented in Table S3.

Initial unadjusted association analyses identified two SNPs that associated with GC under a dominant model where the minor allele was used as the referent. The two SNPs, rs1800440 and rs1056836, in cytochrome P450 family 1 subfamily B member 1 (*CYP1B1*), were both significantly associated with GC after FDR adjustment for multiple testing (adjusted q = 0.07 and q = 0.003, respectively) and were not in L.D. (R^2^ = 0.03) (Table 3). In models adjusted for age, sex, bacterial CagA serostatus, wood stove use, and each SNP singly, both the *CYP1B1* SNP rs1800440 (adjusted q = 0.023) and the *CYP1B1* SNP rs1056836 (adjusted q = 0.023) remained significant (Table 3). Additive and recessive genetic models were also run but no SNPs were significant after FDR correction in the univariate analyses (Table S4).

**Table 3:** Associations of single nucleotide polymorphisms (SNPs) with gastric cancer risk under the univariate and multivariate models.

Chr	SNP	Gene	Major allele	Minor allele	MAF	Univariate OR (95% CI)	Univariate q-value	Multivariate* OR (95% CI)	Multivariate q-value*
1	rs1051740	EPHX1	T	C	0.385	0.99 (080, 1.23)	0.937		
2	rs1800440	CYP1B1	A	G	0.09	1.42 (1.08, 1.89)	**0.070**	1.43 (1.04, 1.97)	**0.023**
2	rs1056836	CYP1B1	C	G	0.243	1.48 (1.20, 1.84)	**0.003**	1.33 (1.04, 1.69)	**0.023**
7	rs2066853	AHR	G	A	0.152	0.90 (0.71, 1.14)	0.645		
8	rs15561	NAT1	A	C	0.433	1.10 (0.81, 1.49)	0.803		
8	rs1801280	NAT1	T	C	0.28	0.96 (0.77, 1.19)	0.864		
8	rs1799930	NAT1	G	A	0.19	0.99 (0.79, 1.23)	0.937		
8	rs1799931	NAT1	G	A	0.128	1.17 (0.92, 1.50)	0.493		
10	rs2031920	CYP2E1	C	T	0.14	1.19 (0.93, 1.52)	0.493		
15	rs762551	CYPIA2	A	C	0.239	1.13 (0.92, 1.40)	0.493		

Univariate analysis N = 1440, multivariable N = 1284.

The referent allele is minor allele.

*Chr* chromosome, *MAF* minor allele frequency.

FDR significant q-values are bolded.

*Adjusted for age, sex, CagA serostatus and wood cookstove use.

We used three statistical models of increasing complexity to assess patterns of association (Table S5). The first multivariable model included the core variables age, sex, wood stove use, bacterial CagA serostatus, and the two *CYP1B1* SNPs. All variables were significant (Table 4). Multivariable model 2 included the core variables, rs1800440, and the interaction of wood stove use and rs1800440. All variables remained significant except for wood stove use and rs1800440 (Table 4). Multivariable model 3 included the same variables as multivariable model 2 and also included SNP rs1056836 with all variables remaining significant except for wood stove use and rs1800440 (Table 4). In both models 2 and 3, the interaction term was statistically significant. The likelihood ratio test identified model 3 with the interaction of wood stove use and rs1800440 as the best model (p = 0.025), and it was used for interpretation. Remarkably, the odds ratio of the interaction term in this model was of similar magnitude as the highly associated bacterial CagA serostatus.

**Table 4:** Associations of multivariable models including interaction terms with gastric cancer.

Characteristics	Multivariable model 1 OR (95% CI)^a^	Multivariable model 1 p-value	Multivariable model 2 OR (95% CI)^a^	Multivariable model 2 p-value	Final multivariable model (3) OR (95% CI)^a^	Final multivariable model (3) p-value
Age	1.04 (1.03, 1.05)	** < 2.00 × 10**^**–16**^	1.04 (1.03, 1.05)	** < 2.00 × 10**^**–16**^	1.04 (1.03, 1.05)	** < 2.00 × 10**^**–16**^
Sex	1.99 (1.55, 2.55)	**5.41 × 10**^**–8**^	1.99 (1.56, 2.55)	**4.89 × 10**^**–8**^	1.98 (1.55, 2.55)	**5.70 × 10**^**–8**^
Wood stove use	2.26 (1.57, 3.29)	**1.70 × 10**^**–5**^	0.95 (0.41, 2.29)	0.91	0.89 (0.39, 2.55)	0.80
Bacterial CagA serostatus	3.47 (2.38, 5.14)	**2.32 × 10**^**–10**^	3.45 (2.37, 5.12)	**2.44 × 10**^**–10**^	3.44 (2.36, 5.11)	**3.14 × 10**^**–10**^
rs1800440	1.57 (1.14, 2.19)	**0.007**	0.57 (0.24, 1.41)	0.21	0.59 (0.25, 1.47)	0.25
rs1056836	1.41 (1.10, 1.80)	**0.007**	NA	NA	1.42 (1.11, 1.82)	**0.006**
Wood stove use:rs1800440	NA	NA	2.93 (1.11, 7.52)	**0.027**	3.05 (1.16, 7.81)	**0.021**

Multivariable model 1 variables: age, sex, wood stove use, bacterial CagA serostatus, rs1800440, and rs1056836.

Multivariable model 2 variables: age, sex, wood stove use, bacterial CagA serostatus, rs1800440, and the interaction of wood stove use with rs1800440.

Multivariable model 3 variables: age, sex, wood stove use, bacterial CagA serostatus, rs1800440, the interaction of wood stove use with rs1800440, and rs1056836.

*95% CI* 95% confidence interval.

p-values ≤ 0.05 are bolded.

^a^Study N = 1284.

**Figure 1:**
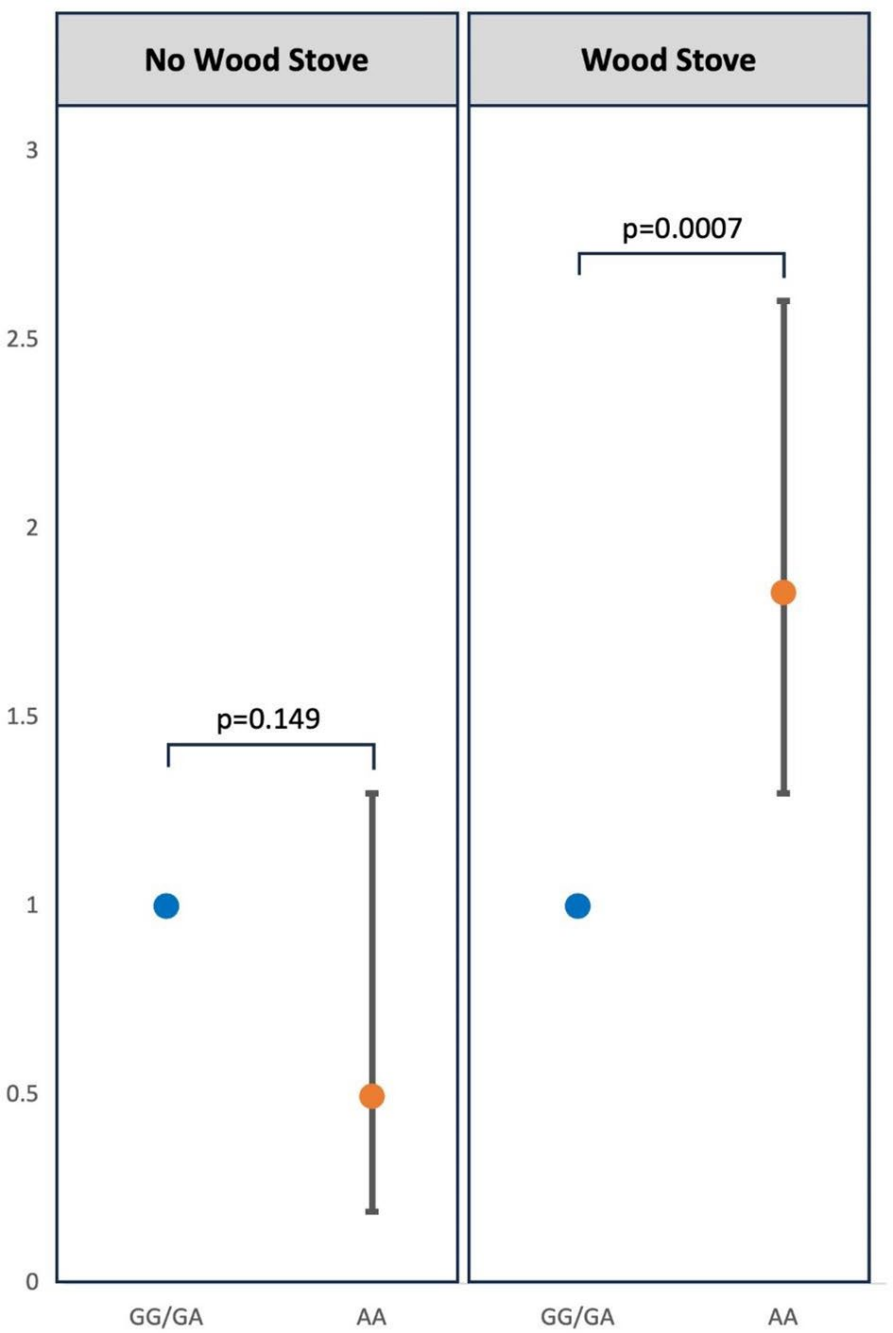
Interaction between wood stove use and the rs1800440 *CYP1B1* SNP. The effect of the rs1800440 AA genotype is dependent on wood stove use. Those that have the rs1800440 AA genotype and use a wood stove have an increased risk of gastric cancer compared to those with the rs1800440 GG or GA genotype and use a wood stove (OR = 1.83; 1.29, 2.60); p = 0.0007). The results derived from Model 3 (Table 5).

Our results showed that genotype did not associate with GC risk for individuals who did not use a wood stove (p = 0.25, OR = 0.59). Nor did wood stove use associate with risk for individuals with GG/GA genotypes (p = 0.80, OR = 0.90). However, wood stove use was associated with GC risk in AA individuals compared to AA individuals who did not use a wood stove (p < 0.0001, OR = 2.73). Finally, for individuals who used a wood stove, AA genotype individuals were at increased risk (p = 0.0007, OR = 1.83) whereas in the absence of wood stove use the same genotype did not associate with GC (Fig. 1; Table 5). Similarly, wood stove use was not associated with GC for people who were GG/GA (Table S6). Multivariable models for rs105836, including the interaction of rs1056836 and wood stove showed no significant interaction between this SNP and wood stove use (Table S7). None of the remaining eight SNPs showed any interactions with woodstove use (Table S8).

**Table 5:** Associations of multivariable models with gastric cancer stratified by wood stove use.

Characteristics	No wood stove (N = 184)		Wood stove (N = 1100)	
	OR (95% CI)^a^	p-value	OR (95% CI)^a^	p-value
Age	1.054 (1.031, 1.080)	< 0.0001	1.036 (1.027, 1.045)	< 0.0001
Sex	2.568 (1.223, 5.609)	0.0147	1.944 (1.494, 2.534)	< 0.0001
CagA serostatus	3.759 (1.407, 12.067)	0.0140	3.350 (2.231, 5.121)	< 0.0001
rs1800440	0.498 (0.191, 1.300)	0.1495	1.833 (1.296, 2.605)	0.0007
rs1056836	0.853 (0.410, 1.769)	0.6694	1.528 (1.173, 1.993)	0.0017

## Discussion

In the rural Central America Four region, we found a significant association between wood cookstove use and gastric adenocarcinoma. Wood stove use increased the risk of GC by more than 2.3 times after adjusting for demographic and risk factors (age, sex, CagA serostatus). This association was independent of the CagA genotype, the principal *H. pylori* virulence and GC risk factor. Importantly, the wood stove use association was significantly modified by the rs1800440 variant of *CYP1B1*, a gene involved in HCA and PAH metabolism, a determinant of the “internal dose”. This SNP was only significant in the context of wood cookstove use, and neither wood stove use nor the SNP were significant when the interaction of this SNP and wood stove use was included in the model, implying strong biological mediation by genotype. Specifically, the rs1800440 AA genotype was high risk when wood stoves were used, but not otherwise, and wood stoves were only significant in the presence of this genotype. These two risk factors are highly prevalent in Central American populations.

The gene-environment interaction between wood stove use and genetics greatly informs our understanding with respect to who is most susceptible to adverse effects of cooking with a wood stove. Individuals who use a wood stove are at an increased risk of developing gastric cancer when they are homozygous for the major rs1800440 allele, whereas they do not appear to be at increased risk in the presence of a minor allele. Similarly, those who do not use a wood stove do not have a significant association based on genotype. This finding supports the concept that elevated risk associated with wood stove use may be mediated by HCA and PAH exposure generated in food preparation with wood stove use and is consistent with the recognized link between meat consumption and gastric and colon cancers. Latin American populations have a high prevalence of the rs1800440 risk allele frequency (86%-88%), as do African and Asian populations with 100% and 99% prevalence, respectively^38^.

Studies examining the rs1800440 *CYP1B1* polymorphism and cancer risk have been inconclusive. The interaction of exposures, such as wood stove use and the variant, may explain the discordant literature related to *CYP1B1* genotypes^39–50^. Our study found that the common variant of the phase I *CYP1B1,* rs1800440, conferred approximately 40% increased risk of gastric cancer even after adjusting for age, sex, CagA status and wood stove use in analyses without a SNP-wood stove interaction term. However, we also noted that the rs1800440 SNP is only significant in the presence of wood stove exposure, a potential explanation of why some prior studies did not find any association between *CYP1B1* rs1800440 and gastric cancer^39^. In colorectal cancer studies, a meta-analysis found no association between colorectal cancer and rs1800440; however, there was considerable heterogeneity among the studies^36^. Carriers of the minor allele have been shown to confer either decreased risk of colorectal cancer, increased risk, or no risk in different studies^41,42^. Interestingly, in a Chinese population, the rs1800440 genotype AG associated with decreased hepatocellular carcinoma risk compared to the AA (homozygous major allele) genotype (adjusted OR = 0.33) when adjusting for similar variables to our study as well as smoking^51^.

Importantly, the rs1800440 *CYP1B1* minor allele (G)* e*ncodes an amino acid substitution Asn453Ser with Ser being likely deleterious with respect to protein function according to Polyphen-2 (score = 0.906) (Fig. S2)^52^. This is consistent with a functional allele increasing internal doses of carcinogens, as in the case of the AA genotypes associating with risk in wood stove users. Our analyses showing a significant interaction between genotype and wood stove use may explain the inconsistencies as prior evidence also indicated that this phase I enzyme minor allele polymorphism may degrade more rapidly than the wild type enzyme and have a drastically shorter half-life, and therefore reduced metabolic activation of estrogens, PAHs, and HCAs^48^.

Limited studies have examined the relationship between wood cookstove use and cancer, and specifically gastric cancer. Compounds generated by wood stoves are modified by metabolizing enzymes, and therefore an interaction between genes for these enzymes is not unexpected. The limited literature indicates that exposure to biomass burning and wood stove cooking has been linked to a variety of cancers of the upper aero-digestive tract, including lung, gastric and esophageal cancer^24,53–62^. The use of wood stoves, diets low in fruits and vegetables, and higher elevation households have all been noted as significant risk factors in an epidemiologic profile of GC in Peru^24^ and the association between cancers of the upper aero-digestive tract was confirmed in southern Brazil after adjusting for tobacco, alcohol, or dietary factors^53^. In addition, the use of biomass fuels was linked to gastrointestinal cancers, including esophageal cancer and gastric cancer, in a large Iranian cohort of men and women^62^. However, how these factors interact with enzymes that activate and deactivate carcinogens has not to our knowledge been studied previously.

Regular wood stove use also contribute to gastric cancer risk through the generation of PAHs. An association has been detected between biomass smoke exposure and gastric cancer, as well as biomass smoke exposure and urinary 1-hydroxypyrene (1-OHP), a PAH metabolite, with a significant trend for dose in a study from Zambia. However, no association was detected between the metabolite and gastric cancer status^61^. Notably, the exposures in sub-Saharan Africa may be less than Latin America as the cook stoves are typically outdoors and often involve the boiling of meats and foods. A recent study of Shanghai women found that women diagnosed with gastric cancer had higher urinary levels of 1-hydroxypyrene glucuronide (1-OHPG), a closely related PAH metabolite^63^.

There is considerable potential for mitigation with improved cookstove design. The Global Alliance for Clean Cook stoves has proposed deployment of 100 million improved stoves^64^. Various designs and interventions have been implemented for the past two decades in order to decrease smoke exposure and deforestation, yet exposure to organic carcinogens in well-done meat and foods cooked at high temperature remain a challenge. Populations with a high prevalence of the risk rs1800440 genotype may benefit most from reduced wood stove use or a changes in food preparation.

The strengths of our study include the regional approach in a rural LMIC population with a high gastric cancer incidence, endemic *H. pylori* cagA infection, and a homogeneous diet. Not having precise quantification of HCA and PAH exposures was one of the study limitations. We did not examine dietary information and meat intake to further quantify HCA and PAH exposure, but this potential influence is dampened by the generally uniform diet in this rural region over time. Lastly, our asymptomatic population-based controls did not undergo endoscopy, and a limited number of control subjects may have had precancerous conditions or early gastric cancer, but this would only decrease the power to detect associations.

## Conclusions

Wood cookstove use contributes to gastric cancer risk in the high-incidence regions of mountainous Central America, where *H. pylori* cagA infection is endemic. We found that the effect is mediated by host genetics, specifically the *CYP1B1* genotype, wherein wood cookstove usage increases risk only in individuals genetically predisposed, to thereby increase the conversion of pro-carcinogenic compounds to carcinogens. Further studies are indicated, as our results underscore the broad health and environmental impact of biomass cookstove use.

### Supplementary Information

Supplementary Information.

## Data Availability

The datasets generated and/or analyzed during the current study are available in the Mendeley Data repository, [Morgan, Douglas; Norwood, Dalton (2023), “Wood cookstove use is associated with gastric cancer in Central America, modified by the CYP1B1 genotype rs1800440, and independent of H. pylori cagA serostatus”, Mendeley Data, V1, https://doi.org/10.17632/mrrjjp4j2w.1].
