# Supplementary material for: Wood cookstove use is associated with gastric cancer in Central America and mediated by host genetics
Source: Sci Rep. 2023 Oct 2;13:16515. doi: 10.1038/s41598-023-42973-7 (PMC10545771; doi:10.1038/s41598-023-42973-7)
Supplement: Supplementary file 1 — Supplementary Information. [file 41598_2023_42973_MOESM1_ESM.docx]

Wood cookstove use is associated with gastric cancer in Central America and mediated by host genetics

**Supplemental Materials**

**Supplemental Table S1. Hypothesis-based single nucleotide polymorphisms (SNPs) analyzed.**

| **Chromosome** | **Position** | **Gene** | **rsID** | **MAF** | **Minor allele** | **Major Allele** |
| --- | --- | --- | --- | --- | --- | --- |
| 1 | 225831932 | EPHX1 | rs1051740 | 0.385 | C | T |
| 2 | 38071060 | CYP1B1 | rs1056836 | 0.243 | G | A |
| 2 | 38070996 | CYP1B1 | rs1800440 | 0.090 | A | G |
| 2 | 233682280 | UGTIA7 | rs61261057* | 0.003 | G | C |
| 7 | 17339486 | AHR | rs2066853 | 0.152 | A | G |
| 8 | 18400285 | NAT1 | rs1041983 | 0.323 | A | C |
| 8 | 18400806 | NAT1 | rs1208 | 0.302 | A | G |
| 8 | 18223142 | NAT1 | rs15561 | 0.433 | T | C |
| 8 | 18400484 | NAT1 | rs1799929 | 0.287 | C | T |
| 8 | 18400593 | NAT1 | rs1799930 | 0.190 | T | C |
| 8 | 18400194 | NAT1 | rs1801279* | 0.002 | A | G |
| 8 | 18400860 | NAT1 | rs1799931 | 0.128 | G | A |
| 8 | 18400344 | NAT1 | rs1801280 | 0.280 | A | G |
| 10 | 133526341 | CYP2E1 | rs2031920 | 0.140 | T | C |
| 15 | 74749576 | CYPIA2 | rs762551 | 0.239 | C | A |

**Notes**

• MAF, Minor allele frequency

• *****SNP removed from final analysis: MAF < 0.05

• The reference genome for Position was obtained from (GRCh38.p14).

**Supplemental Table S2. Single nucleotide polymorphisms (SNPs) analyzed, final primer design.**

| **Variant (alleles)** | **Forward Primer Sequences** | **Reverse Primer Sequences** |
| --- | --- | --- |
| rs1051740 | ACG TTG GAT GCT GGC GTT TTG CAA ACA TAC | ACG TTG GAT GTG GAA GAA GCA GGT GGA GAT |
| rs1056836 | ACG TTG GAT GCT GTG GTT TTT GTC AAC CAG | ACG TTG GAT GAA AGT TCT CCG GGT TAG GC |
| rs1800440 | ACG TTG GAT GAA AAA TCA TCA CTC TGC TGG | ACG TTG GAT GTC GAT TCT TGG ACA AGG A |
| rs61261057* | ACG TTG GAT GCA TTG CGA AGT GCA TTT TCT C | ACG TTG GAT GCA AAC TCC TGC AAT TTG AAA |
| rs2066853 | ACG TTG GAT GCA CCA GAA AAA TCA TTT CTG A | ACG TTG GAT GCC TAG GCA TTG ATT TTG AAG |
| rs1041983 | ACG TTG GAT GCC ATG CCA GTG CTG TAT TTG | ACG TTG GAT GAC CAC AAT CGG TTT TCA GAC |
| rs1208 | ACG TTG GAT GTT TGG GCA CGA GAT TTC TCC | ACG TTG GAT GTC TCA CTG AGG AAG AGG TTG |
| rs15561 | ACG TTG GAT GAG AAA CAT AAC CAC AAA CC | ACG TTG GAT GCA AGA TAA CCA CAG GCC ATC |
| rs1799929 | ACG TTG GAT GTG TAA TAT ACT GCT CTC TCC | ACG TTG GAT GCC TTG CAT TTT CTG CTT GAC |
| rs1799930 | ACG TTG GAT GCC TGC CAA AGA AGA AAC ACC | ACG TTG GAT GTC ATA GAC TCA AAA TCT TC |
| rs1801279* | ACG TTG GAT GTT GAT TGA CCT GGA GAC ACC | ACG TTG GAT GCC ATG GAG TTG GGC TTA GAG |
| rs1799931 | ACG TTG GAT GAA ATC TCG TGC CCA AAC CTG | ACG TTG GAT GGG GTG ATA CAT ACA CAA GGG |
| rs1801280 | ACG TTG GAT GCA TGG TTC ACC TTC TCC TGC | ACG TTG GAT GTC TGG GAG GAG CTT CCA GAC |
| rs2031920 | ACG TTG GAT GGT TCT TAA TTC ATA GGT TGC | ACG TTG GAT GCA TTT CTC ATC ATA TTT TC |
| rs762551 | ACG TTG GAT GCA GCT GGA TAC CAG AAA GAC | ACG TTG GAT GAT TCT GTG ATG CTC AAA GGG |

**Notes**

• All sequencing was performed on the Sequenom platform in the Vanderbilt Technologies for Advanced Genomics (VANTAGE) core laboratory of Vanderbilt University Medical Center (VUMC).

**Supplemental Table S3. Genotype counts by case-control status**

| Chr^§^ | SNP | Gene | Genotype | Control | Cases |
| --- | --- | --- | --- | --- | --- |
| 1 | rs1051740 | EPHX1 | TT | 298 (36.5%) | 251 (36.9%) |
|  |  |  | TC | 418 (51.2%) | 327 (48.0%) |
|  |  |  | CC | 100 (12.3%) | 103 (15.1%) |
| 2 | rs1800440 | CYP1B1 | AA | 659 (80.8%) | 582 (85.3%) |
|  |  |  | GA | 146 (17.9%) | 96 (14.1%) |
|  |  |  | GG | 11 (1.3%) | 4 (0.6%) |
| 2 | rs1056836 | CYP1B1 | CC | 434 (53.2%) | 424 (62.7%) |
|  |  |  | GC | 337 (41.3%) | 205 (30.3%) |
|  |  |  | GG | 45 (5.5%) | 47 (7.0%) |
| 7 | rs2066853 | AHR | GG | 582 (72.1%) | 498 (73.2%) |
|  |  |  | GA | 200 (24.8%) | 164 (24.1%) |
|  |  |  | AA | 25 (3.1%) | 18 (2.6%) |
| 8 | rs15561 | NAT1 | CC | 250 (30.9%) | 223 (32.7%) |
|  |  |  | CA | 412 (50.9%) | 339 (49.7%) |
|  |  |  | AA | 148 (18.3%) | 120 (17.6%) |
| 8 | rs1801280 | NAT1 | TT | 395 (50.8%) | 330 (50.8%) |
|  |  |  | CT | 320 (41.2%) | 281 (43.2%) |
|  |  |  | CC | 62 (8.0%) | 39 (6.0%) |
| 8 | rs1799930 | NAT1 | GG | 543 (66.7%) | 452 (66.7%) |
|  |  |  | GA | 225 (27.6%) | 203 (29.9%) |
|  |  |  | AA | 46 (5.7%) | 23 (3.4%) |
| 8 | rs1799931 | NAT1 | GG | 628 (77.2%) | 502 (74.0%) |
|  |  |  | GA | 176 (21.6%) | 163 (24.0%) |
|  |  |  | AA | 9 (1.1%) | 13 (1.9%) |
| 10 | rs2031920 | CYP2E1 | CC | 606 (76.7%) | 472 (72.7%) |
|  |  |  | TC | 159 (20.1%) | 159 (24.5%) |
|  |  |  | TT | 25 (3.2%) | 18 (2.8%) |
| 15 | rs762551 | CYPIA2 | AA | 481 (59.1%) | 386 (56.7%) |
|  |  |  | CA | 292 (35.9%) | 260 (38.2%) |
|  |  |  | CC | 41 (5.0%) | 35 (5.1%) |

**Supplemental Table S4. Univariate associated analyses for SNPs with MAF > 0.05**

|  |  |  | **Additive Model** | | **Recessive Model** | |
| --- | --- | --- | --- | --- | --- | --- |
| Chr^§^ | SNP | Gene | OR (95% CI) | p-value | OR (95% CI) | p-value |
| 1 | rs1051740 | EPHX1 | 1.06 (0.91, 1.23) | 0.4665 | 1.28 (0.95, 1.72) | 0.1069 |
| 2 | rs1800440 | CYP1B1 | 0.72 (0.56, 0.933 | 0.0126 | 0.43 (0.14, 1.36) | 0.1524 |
| 2 | rs1056836 | CYP1B1 | 0.80 (0.68, 0.95) | 0.0110 | 1.28 (0.84, 1.95) | 0.2514 |
| 7 | rs2066853 | AHR | 0.94 (0.78, 1.15) | 0.5624 | 0.85 (0.46, 1.57) | 0.6056 |
| 8 | rs15561 | NAT1 | 0.95 (0.82, 1.10) | 0.4850 | 0.96 (0.73, 1.25) | 0.7350 |
| 8 | rs1801280 | NAT1 | 0.95 (0.81, 1.13) | 0.5639 | 0.74 (0.49, 1.12) | 0.1478 |
| 8 | rs1799930 | NAT1 | 0.94 (0.78, 1.12) | 0.4576 | 0.59 (0.35, 0.97) | 0.0407 |
| 8 | rs1799931 | NAT1 | 0.83 (0.67, 1.04) | 0.1006 | 0.57 (0.24, 1.35) | 0.2019 |
| 10 | rs2031920 | CYP2E1 | 1.15 (0.94, 1.40) | 0.1856 | 0.87 (0.47, 1.62) | 0.6648 |
| 15 | rs762551 | CYPIA2 | 1.07 (0.91, 1.28) | 0.4136 | 1.02 (0.64, 1.62) | 0.9282 |

**Supplemental Table S5. Logistic Multivariable Models**

| Multivariable model 1 including variables age, sex, wood stove use, bacterial CagA serostatus, rs1800440, and rs1056836  log_b_$\frac{p}{1-p}$ = **β_0_** + β_1_x_1_ + β_2_x_2_ + β_3_x_3_ + β_4_x_4_ + β_5_x_5_ **+** β_6_x_6_  Multivariable model 2 including variables age, sex, wood stove use, bacterial CagA serostatus, rs1800440, and the interaction between wood stove use and rs1800440  log_b_$\frac{p}{1-p}$ = **β_0_** + β_1_x_1_ + β_2_x_2_ + β_3_x_3_ + β_4_x_4_ + β_5_x_5_ **+** β_7_x_3_:x_5_  Multivariable model 3 including variables age, sex, wood stove use, bacterial CagA serostatus, rs1800440, rs1056836, and the interaction between wood stove use and rs1800440  log_b_$\frac{p}{1-p}$ = **β_0_** + β_1_x_1_ + β_2_x_2_ + β_3_x_3_ + β_4_x_4_ + β_5_x_5_ **+** β_6_x_6_ **+** β_7_x_3_:x_5_ |
| --- |

First term indicates model's intercept. The second through sixth term represent the variables age (x1), sex (x2), wood stove use (x3), bacterial CagA serostatus (x4), SNP rs1800440 (x5) and SNP rs1056836 (x6), respectively.

**Supplemental Table S6. Associations of multivariable models with gastric cancer stratified by rs1800440**

|  | **rs1800440 = GG/GA (N=222)** | | **rs1800440 = AA (N=1062)** | |
| --- | --- | --- | --- | --- |
| **Characteristics** | **OR (95% CI)^a^** | **p-value** | **OR (95% CI)^a^** | **p-value** |
| Age | 1.044 (1.023, 1.067) | <0.0001 | 1.037 (1.028, 1.047) | <0.0001 |
| Sex | 2.349 (1.265, 4.424) | 0.0073 | 1.937 (1.478, 2.543) | <0.0001 |
| Wood stove Use | 0.841 (0.343, 2.109) | 0.7071 | 2.731 (1.826, 4.149) | <0.0001 |
| Bacterial CagA serostatus | 6.989 (2.429, 25.814) | 0.0010 | 3.087 (2.060, 4.707) | <0.0001 |
| rs1056836 | 1.650 (0.803, 3.493) | 0.1802 | 1.398 (1.073, 1.822) | 0.0131 |

**Supplemental Table S7. Gastric cancer association in multivariable models with**

**the rs1056836-wood stove interaction terms.**

| **Characteristics** | **Supplemental Multivariable Model 1 OR (95% CI)^a^** | **Supplemental Multivariable Model 1 p-value** | **Supplemental**  **Multivariable Model 2**  **OR (95% CI)^a^** | **Supplemental Multivariable Model 2**  **p-value** |
| --- | --- | --- | --- | --- |
| **Age** | 1.04 (1.03, 1.05) | **< 2.00 x 10^-16^** | 1.04 (1.03, 1.05) | **< 2.00 x 10^-16^** |
| **Sex** | 2.01 (1.57, 2.58) | **2.81 x 10^-8^** | 1.99 (1.55, 2.55) | **5.22 x 10^-8^** |
| **Wood stove Use** | 1.78 (1.06, 3.05) | **0.031** | 1.75 (1.04, 3.00) | **0.037** |
| **Bacterial CagA serostatus** | 3.45 (2.36, 5.11) | **2.51 x 10^-10^** | 3.45 (2.37, 5.12) | **2.73 x 10^-10^** |
| **rs1800440** | NA | NA | 1.59 (1.14, 2.21) | **0.006** |
| **rs1056836** | 0.89 (0.45, 1.78) | 0.75 | 0.92 (0.46, 1.84) | 0.81 |
| **Wood stove Use:rs1056836** | 1.57 (0.7, 3.03) | 0.23 | 1.63 (0.78, 3.44) | 0.20 |

• Supplemental Multivariable Model 1 variables: Age, Sex, Wood stove Use, Bacterial CagA serostatus, rs1800440, and rs1056836.

• Supplemental Multivariable Model 2 variables: Age, Sex, Wood stove Use, Bacterial CagA serostatus, rs1800440, rs1056836, and the interaction of Wood stove Use and rs1056836

**Supplemental Table S8. Woodstove by SNP model including interactions terms and dominant coding for each SNP (Model y = WS + SNP + WS*SNP)**

| Chr^§^ | SNP | Gene | Wood Stove Use  p-value | SNP  p-value | Interaction  p-value |
| --- | --- | --- | --- | --- | --- |
| 1 | rs1051740 | EPHX1 | <0.0001 | 0.8936 | 0.9433 |
| 7 | rs2066853 | AHR | 0.0003 | 0.2190 | 0.2156 |
| 8 | rs15561 | NAT1 | <0.0001 | 0.6948 | 0.9794 |
| 8 | rs1801280 | NAT1 | <0.0001 | 0.0700 | 0.0381* |
| 8 | rs1799930 | NAT1 | <0.0001 | 0.0828 | 0.0545* |
| 8 | rs1799931 | NAT1 | 0.0265 | 0.1112 | 0.2865 |
| 10 | rs2031920 | CYP2E1 | 0.0002 | 0.3276 | 0.0919 |
| 15 | rs762551 | CYPIA2 | 0.0014 | 0.2014 | 0.4130 |

**Notes**: * When added to the Multivariable Models 1, 2, and 3, these SNPs and their interactions with wood stove use was not statistically significant.

**Supplemental Figure S1. Single nucleotide polymorphisms (SNPs) in linkage disequilibrium.**


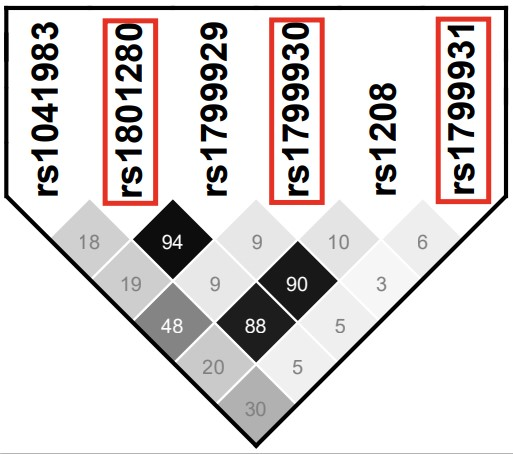


The red box indicates the SNPs kept in the analysis from the NAT1 gene in the final analysis. The removed SNPs had an R^2^ greater than 0.4.

**Supplemental Figure S2. The Asn453 residue prediction and 3D visualization for**

**the rs1800440 *CYP1B1* SNP**


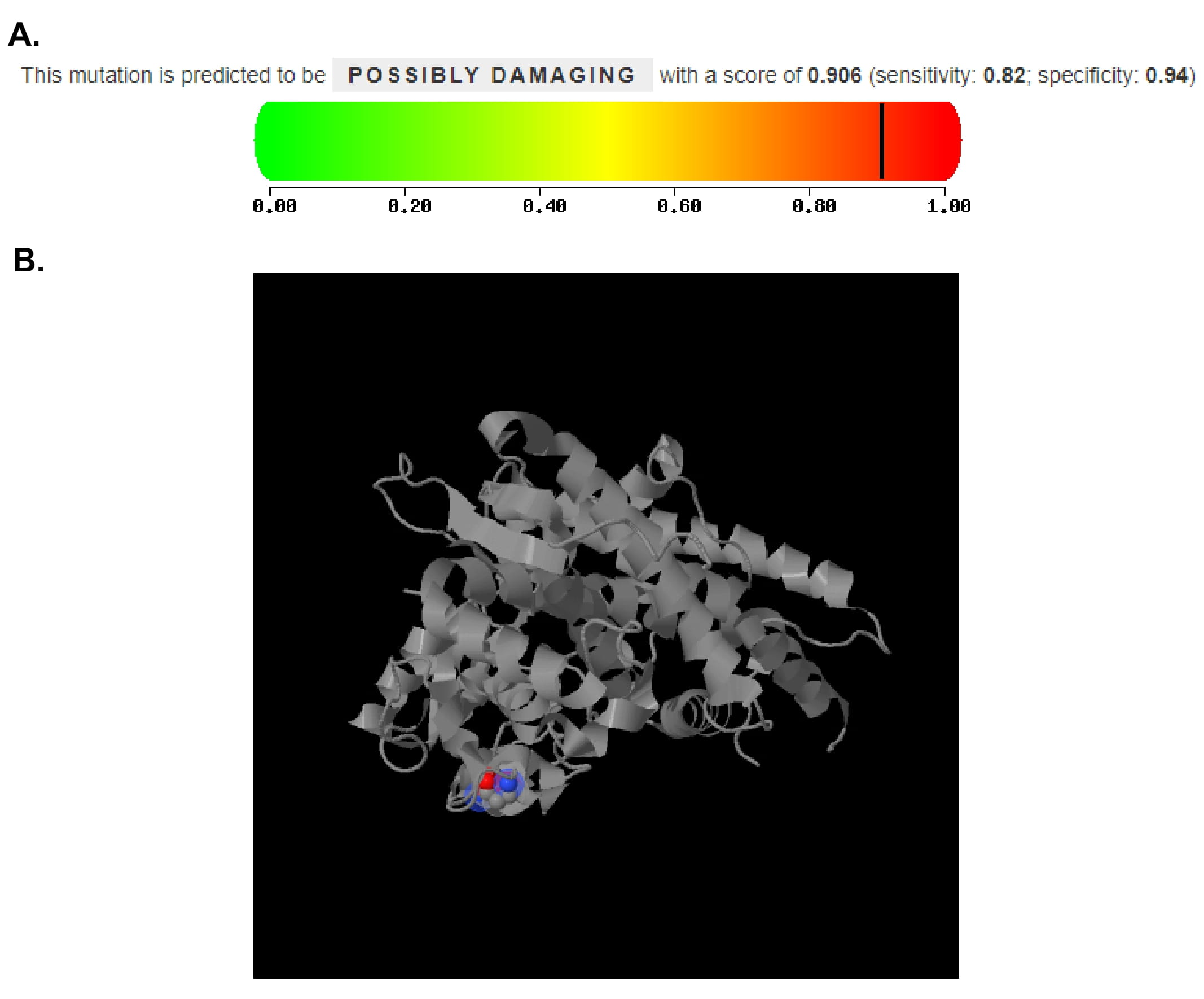


A. Polyphen-2 reports that the rs1800440 *CYP1B1* polymorphism encodes an amino acid substitution N453S with the S amino acid as possibly damaging.

B. The 3D visualization of the Asn453 residue where the mutation is notated as blue and red circles.
